# Subtype-specific sonographic signatures and clinicopathological features of metaplastic breast cancer: a 50-case cohort study

**DOI:** 10.3389/fmed.2026.1849473

**Published:** 2026-07-02

**Authors:** Boxiong Wei, Zijing Fu, Yuhong Shao, Xiuming Sun, Luzeng Chen

**Affiliations:** Department of Ultrasound, Peking University First Hospital, Beijing, China

**Keywords:** BI-RADS, histological subtypes, lymph node metastasis, metaplastic breast cancer, ultrasonography

## Abstract

**Background:**

Metaplastic breast cancer (MBC) is a rare, aggressive malignancy with profound histological heterogeneity. This study aimed to describe the subtype-specific sonographic signatures and baseline clinicopathological features of MBC based on the World Health Organization classification.

**Methods:**

This exploratory single-center retrospective cohort study included 50 women with histologically confirmed MBC (15 spindle cell, 12 squamous cell, and 23 mixed type) treated between January 2010 and January 2026. Preoperative ultrasound features, evaluated via BI-RADS criteria, and baseline clinicopathological parameters were compared across subtypes.

**Results:**

The spindle cell subtype frequently presented with a pseudo-benign sonographic phenotype, characterized by circumscribed margins (60.0%) and oval or round shape (46.7%), along with significantly smaller tumor dimensions (median 3.1 cm; *p* = 0.041). Conversely, squamous cell and mixed-type tumors more often showed heterogeneous echogenicity (83.3 and 73.9%, respectively; *p* = 0.003) and complex cystic-solid components (75.0 and 56.5%, respectively; *p* = 0.037), which may reflect cystic degeneration or necrosis. In addition, a descriptive pattern suggesting size-node dissociation was observed: despite a large mean primary tumor size of 4.7 cm, the overall axillary lymph node metastasis rate was 22.0%, with similar rates in tumors measuring 2–5 cm and >5 cm (26.1 and 23.8%, respectively).

**Conclusion:**

MBC demonstrates significant subtype-specific heterogeneity. The pseudo-benign sonographic appearance of the spindle cell subtype represents a major diagnostic pitfall, supporting prompt biopsy for rapidly enlarging lesions. The observed size-node pattern should be interpreted as descriptive and hypothesis-generating, because nodal events were limited and long-term survival, recurrence, and distant metastasis data were unavailable. Recognizing these distinct patterns may facilitate accurate diagnosis and timely multidisciplinary management.

## Introduction

Metaplastic breast cancer (MBC) is a rare and aggressive disease, accounting for less than 1% of all breast carcinomas (1). Most MBCs exhibit a triple-negative phenotype. Consequently, patients typically experience worse survival and higher recurrence rates compared to those with conventional invasive ductal carcinoma (2, 3). Furthermore, MBC responds poorly to standard neoadjuvant chemotherapy. Recent studies report low pathological complete response rates, often around 10% or lower (4, 5). As a result, early and accurate diagnosis is important for timely biopsy and multidisciplinary treatment planning, including consideration of upfront surgery in selected operable patients (6).

Diagnosing MBC is challenging due to its diverse histology. Based on the latest World Health Organization (WHO) classification, MBC consists of several distinct subtypes, including spindle cell, squamous cell, and mixed type (including matrix-producing components) (1, 7). Because of these varied subtypes, the tumor’s appearance on ultrasound is also highly variable, often lacking classic malignant features (8). Some MBCs—especially the spindle cell subtype—exhibit a “pseudo-benign” morphology with a regular shape and circumscribed margins. This may lead to low-suspicion ultrasound assessment or diagnostic underestimation (9). In some cases, spindle cell tumors may be assigned low-suspicion BI-RADS categories, such as BI-RADS 3 or 4A. BI-RADS 3 may delay biopsy, whereas BI-RADS 4A usually prompts biopsy but still reflects a low level of suspicion (10).

Besides atypical imaging features, MBC also shows an unusual pattern of lymph node metastasis (11). In typical breast cancer, larger tumors are more likely to spread to lymph nodes. However, MBC patients often have large tumors (>5 cm) but negative axillary lymph nodes. Recent analyses of the National Cancer Database have reported a similar pattern of relatively low nodal involvement despite large tumor size (12, 13). One proposed explanation is that epithelial–mesenchymal transition (EMT)-related biology in MBC may favor hematogenous spread over lymphatic spread (14, 15), although this mechanism cannot be confirmed by ultrasound findings alone. Despite these findings, most imaging studies still group all MBC cases together (16). There is limited research comparing the specific ultrasound features and baseline clinicopathological features of different WHO subtypes.

To address this knowledge gap, the present study retrospectively analyzed 50 histologically confirmed MBC cases strictly stratified by WHO subtypes (spindle cell, squamous cell, and mixed). We aimed to describe the distinct sonographic signatures and baseline clinicopathological features of each subtype. By identifying these subtype-specific patterns, this study seeks to provide radiologists with practical diagnostic clues for early recognition of these deceptive tumors, and to support multidisciplinary teams in optimizing surgical planning.

## Materials and methods

### Study design and ethics approval

This retrospective cohort study investigated the sonographic and clinicopathological features of metaplastic breast cancer. Patient data were collected at Peking University First Hospital from January 2010 to January 2026. Ethical approval was granted by the hospital’s Institutional Review Board. Given the retrospective design and the use of routinely collected medical records, the need for individual written informed consent was formally waived. The study was conducted in accordance with standard national ethical guidelines for human research. This was an exploratory retrospective cohort study rather than a confirmatory prognostic analysis. Because MBC is extremely rare, no formal *a priori* sample size calculation was performed; instead, all eligible consecutive patients treated during the study period were included. Given the long inclusion period from 2010 to 2026, potential temporal heterogeneity in ultrasound equipment, imaging protocols, and clinical practice was addressed by retrospective re-evaluation of all stored images and cine loops by two breast ultrasound specialists using the standardized ACR BI-RADS 5th edition lexicon. Patients who had received neoadjuvant therapy before baseline ultrasound were excluded.

### Patient selection

We identified 50 female patients with biopsy- or surgically confirmed metaplastic breast cancer from the medical and pathology records at Peking University First Hospital. The inclusion criteria were defined as follows: (1) a definitive histopathological diagnosis of MBC, subtyped according to the WHO classification of Breast Tumours; (2) availability of complete, high-quality preoperative breast ultrasonographic examination data; and (3) complete baseline clinical and pathological records (including tumor size, lymph node status, and immunohistochemical markers). Patients with factors that could alter or confound the natural ultrasonographic findings of the tumors were strictly excluded. Specifically, any patients who received neoadjuvant chemotherapy, targeted therapy, or local radiation prior to their baseline ultrasound assessment were excluded, as these treatments significantly distort tumor morphology. Furthermore, patients presenting with concurrent independent ipsilateral breast malignancies (such as a separate invasive ductal carcinoma) or those with degraded imaging data that precluded accurate morphological evaluation were also excluded from the final cohort. All eligible consecutive patients during the study period were included to minimize selection bias.

### Ultrasonographic examination

Preoperative breast ultrasonography was performed using various advanced high-resolution ultrasound platforms, including the GE LOGIQ E8, E9, E10, Philips EPIQ 7, Elite, Canon Aplio i800, and Mindray Resona R9 equipped with 5–14 MHz high-frequency linear-array transducers. To account for the use of different equipment during the study period, all image acquisitions strictly adhered to standardized institutional protocols to minimize inter-device variability. The examinations were performed before final pathological confirmation was available. All stored images and cine loops were subsequently re-evaluated by dedicated breast ultrasound specialists who were blinded to the patients’ clinical histories, treatment details, and final pathological subtypes. Both gray-scale and color Doppler imaging modalities were utilized for every breast lesion. The gray-scale analysis systematically evaluated the mass size, shape, margin, orientation, internal echogenicity (specifically noting any complex cystic-solid changes or calcifications), in accordance with the standardized criteria of the American College of Radiology (ACR) BI-RADS. Additionally, color Doppler imaging was routinely applied to assess the internal and peripheral blood flow signals of the tumors.

### Ultrasonographic evaluation

To ensure an objective assessment, all stored static ultrasound images and real-time cine loops were retrospectively analyzed by two dedicated breast ultrasound specialists with 6 and 10 years of clinical experience, respectively. Both readers evaluated the images independently and were completely blinded to the patients’ clinical histories, surgical details, and final pathological subtypes. Any disagreements between the two reviewers were resolved through direct discussion to reach a consensus. If an agreement could not be reached, a third senior attending physician with over 20 years of experience provided the final adjudication.

The sonographic characteristics of each tumor were categorized strictly according to the 5th edition of the ACR BI-RADS guidelines. The evaluated gray-scale parameters included the maximum tumor diameter, shape (oval, round, or irregular), margin (circumscribed or not circumscribed), and orientation (parallel or not parallel to the skin). Given the histological heterogeneity of MBC, the reviewers paid particular attention to internal echo patterns. They documented the presence of complex cystic-solid or anechoic components, which may correspond to cystic degeneration or necrosis, and echogenic foci, which may correspond to calcifications or osseous/chondroid matrix components.

For color Doppler evaluation, the internal vascularity of the masses was subjectively assessed and graded based on the Adler criteria (grades 0 to III). Finally, the ipsilateral axillary lymph nodes were carefully evaluated. A lymph node was classified as suspicious for metastasis if it demonstrated cortical thickening greater than 3 mm, a loss of the normal echogenic fatty hilum, or an abnormal rounded morphology. Axillary lymph node metastasis in the clinicopathological analysis was defined by final pathological nodal status when axillary surgery or nodal biopsy was available. Sonographically suspicious lymph nodes were recorded separately and were not considered metastatic unless pathologically confirmed.

For this study, a “pseudo-benign” sonographic appearance was defined as a solid lesion showing at least two of the following three benign-appearing BI-RADS descriptors: oval or round shape, circumscribed margin, and parallel orientation. This term refers only to imaging morphology and does not imply a benign pathological diagnosis. Initial diagnostic underestimation was defined as an original ultrasound assessment of BI-RADS 3 or 4A in a lesion later confirmed as MBC.

### Pathological reference standard

All histopathological diagnoses served as the reference standard for this study. The final pathological evaluation was primarily based on surgically excised specimens, which allowed for a comprehensive assessment of the tumors’ highly heterogeneous components. For patients who did not undergo surgery, ultrasound-guided core needle biopsy results were utilized. The tumors were thoroughly reviewed by two experienced breast pathologists and categorized into specific subtypes (spindle cell, squamous cell, and mixed type) in strict accordance with the 5th edition of the WHO classification of Breast Tumours.

Furthermore, immunohistochemical staining was routinely performed on the formalin-fixed, paraffin-embedded tissue blocks. Estrogen receptor (ER) and progesterone receptor (PR) negativity were strictly defined as less than 1% positive nuclear staining. Human epidermal growth factor receptor 2 (HER2) status was evaluated according to the latest American Society of Clinical Oncology/College of American Pathologists guidelines. Tumors lacking ER, PR, and HER2 expression were classified as the triple-negative phenotype. The Ki-67 proliferation index was also recorded as the percentage of positively stained invasive tumor cells.

### Statistical analysis

Data processing and statistical evaluations were carried out using IBM SPSS Statistics for Windows, version 27.0 (Armonk, NY). The normality of continuous variables was assessed using the Shapiro–Wilk test. Normally distributed data are presented as means ± standard deviations (SD) and were compared among the three pathological subtypes using one-way analysis of variance (ANOVA). For skewed continuous data, medians with interquartile ranges (IQR) are reported, with overall group comparisons performed via the Kruskal–Wallis *H* test. Categorical parameters are summarized as absolute counts and percentages, and overall differences among the three subtypes were assessed using Pearson’s chi-square test or Fisher’s exact test, as appropriate.

When an overall comparison showed a significant difference among the three subtypes, pairwise *post-hoc* comparisons were performed with Bonferroni correction. For categorical variables, Fisher’s exact test was used when appropriate. Exact adjusted *p*-values were reported. Effect sizes were also reported when appropriate, including Cramer’s *V* for categorical variables. Multivariable regression was not performed because only 11 patients had axillary lymph node metastasis. To provide a limited formal assessment of the size-node pattern, nodal metastasis rates were compared among the three tumor-size categories (≤2 cm, 2–5 cm, and >5 cm) using the Fisher–Freeman–Halton exact test. A linear trend test was also performed because the categories were ordered. With so few events, adjustment for several covariates would be unstable and could cause overfitting. Therefore, all subgroup analyses were treated as exploratory and unadjusted. A two-sided *p-*value of < 0.05 was considered statistically significant. Disagreements between readers were resolved by consensus.

## Results

### Patient characteristics

The study cohort consisted of 50 women diagnosed with metaplastic breast cancer. The mean age at diagnosis was 55.3 ± 11.5 years, and 32 patients (64.0%) were postmenopausal. When stratified by pathological subtype, no significant differences were observed in age (*p* = 0.682) or menopausal status (*p* = 0.892) among the squamous cell (*n* = 12), spindle cell (*n* = 15), and mixed type (*n* = 23) groups, suggesting no obvious imbalance in these baseline variables (Table 1).

**Table 1:** Baseline clinical and clinicopathological characteristics of 50 MBC patients.

Characteristic	Overall (*n* = 50)	Squamous cell (*n* = 12)	Spindle cell (*n* = 15)	Mixed type (*n* = 23)	*p*-value
Patient demographics					
Age (years), mean ± SD	55.3 ± 11.5	55.5 ± 10.8	53.5 ± 10.5	56.5 ± 12.5	0.682^a^
Postmenopausal, *n* (%)	32 (64.0%)	7 (58.3%)	10 (66.7%)	15 (65.2%)	0.892^b^
Clinical features					
Palpable mass, *n* (%)	46 (92.0%)†	11 (91.7%)	14 (93.3%)	21 (91.3%)	1.000^c^
Localized pain or tenderness, *n* (%)	16 (32.0%)	6 (50.0%)	3 (20.0%)	7 (30.4%)	0.246^b^
Rapid tumor growth, *n* (%)	25 (50.0%)	7 (58.3%)	7 (46.7%)	11 (47.8%)	0.801^b^
Pathological parameters					
Tumor diameter (cm), mean ± SD	4.7 ± 2.6	4.5 ± 1.2	3.4 ± 2.8	5.6 ± 2.7	—
Tumor diameter (cm), median (IQR)	4.2 (3.0–6.0)	4.4 (3.7–5.2)	3.1 (2.0–4.6)	5.4 (4.0–7.0)	0.041^d,*^
High histologic grade (Grade III), *n* (%)	42 (84.0%)	9 (75.0%)	13 (86.7%)	20 (87.0%)	0.623^c^
Ki-67 index (%), mean ± SD	50.9 ± 22.0	60.0 ± 21.0	40.5 ± 17.5	53.0 ± 22.5	0.052^a^
Axillary lymph node metastasis, *n* (%)	11 (22.0%)	4 (33.3%)	2 (13.3%)	5 (21.7%)	0.514^c^
Molecular subtypes					
Triple-negative phenotype, *n* (%)	44 (88.0%)	9 (75.0%)	14 (93.3%)	21 (91.3%)	0.393^c^
Luminal type (ER or PR positive), *n* (%)	3 (6.0%)	1 (8.3%)	1 (6.7%)	1 (4.3%)	1.000^c^
HER2-positive type, *n* (%)	3 (6.0%)	2 (16.7%)	0 (0.0%)	1 (4.3%)	0.227^c^

MBC, metaplastic breast cancer; SD, standard deviation; IQR, interquartile range.

^†^Majority of patients presented with palpable masses, consistent with typical symptomatic presentation of MBC (small proportion non-palpable, potential selection bias acknowledged).

^a^One-way ANOVA; ^b^Chi-square test; ^c^Fisher’s exact test; ^d^Kruskal–Wallis *H* test. *p < 0.05. For significant continuous variables, *post-hoc* pairwise comparisons were performed using Bonferroni correction. Exact adjusted *p*-values are reported in the Results section.

Clinically, the vast majority of patients (92.0%, 46/50) presented with a palpable breast mass. Localized pain or tenderness was reported in 16 cases (32.0%) overall. Although localized pain or tenderness appeared more frequent in the squamous cell subgroup (50.0%) compared to the spindle cell subgroup (20.0%), the difference was not statistically significant (*p* = 0.246). Rapid tumor growth was reported in 50.0% (25/50) of the cohort, with no significant difference observed among the subtypes (*p* = 0.801).

### Tumor pathology

Histologically, the cohort was characterized by aggressive features, with 84.0% (42/50) of tumors classified as high grade (Grade III). The triple-negative phenotype was the dominant molecular subtype, identified in 88.0% (44/50) of patients, while a small subset (6.0%, 3/50) exhibited Luminal (ER or PR positive) features.

**Figure 1:**
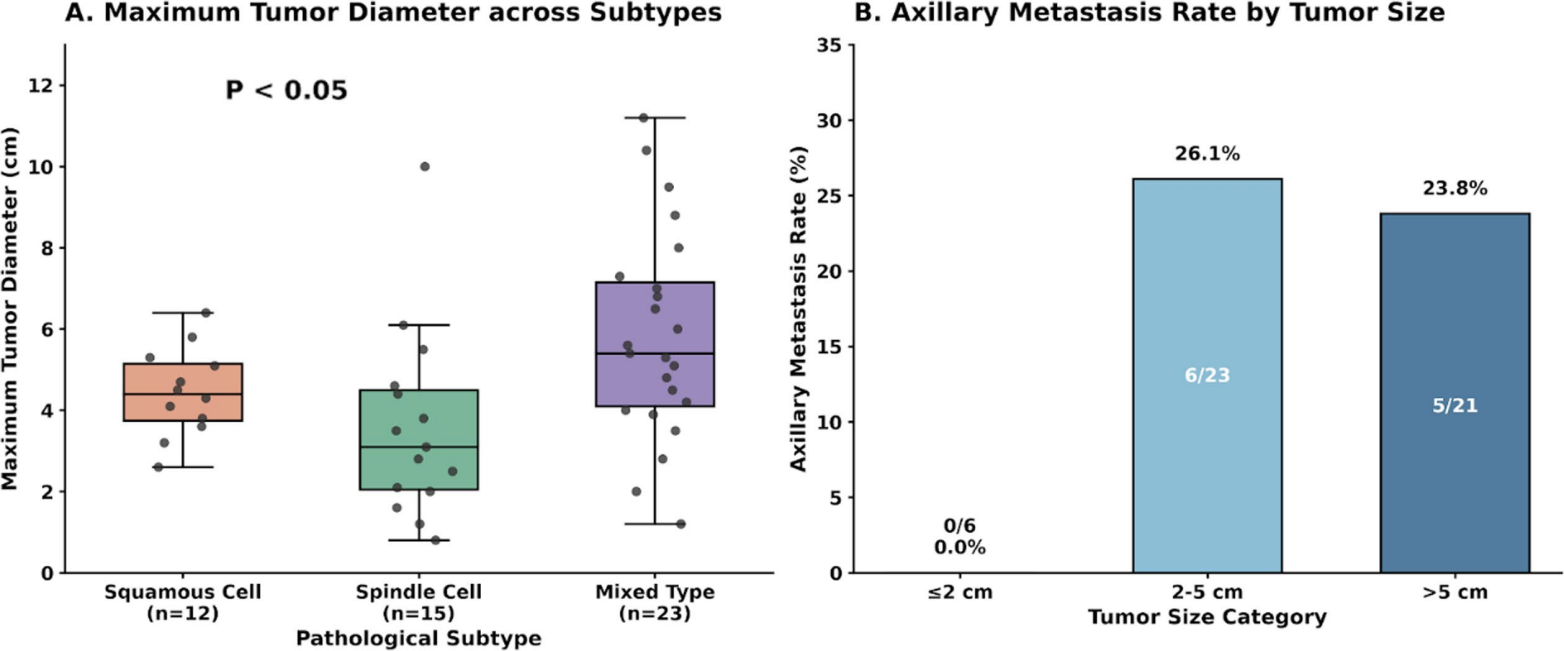
Tumor diameter by pathological subtype **(A)** and nodal metastasis rate by tumor size group **(B)**. **(A)** Box-and-whisker plots show the distribution of maximum tumor diameter. The overall difference among subtypes was significant (Kruskal–Wallis *H* test, *p* = 0.041). *Post-hoc* testing showed a significant difference only between the spindle cell and mixed-type groups (adjusted *p* = 0.036). **(B)** The nodal metastasis rate was 0.0% in tumors ≤2 cm, 26.1% in tumors measuring 2–5 cm, and 23.8% in tumors >5 cm. The difference across tumor-size groups was not statistically significant (Fisher–Freeman–Halton exact test, *p* = 0.563), and no significant linear trend was detected (*p* = 0.387).

Differences in tumor size were observed among the subtypes, while Ki-67 did not differ significantly, although the spindle cell group showed a numerically lower mean value (Figure 1A). The mixed type presented with the largest median tumor diameter (5.4 cm, IQR: 4.0–7.0 cm), followed by squamous cell carcinoma (4.4 cm, IQR: 3.7–5.2 cm). In contrast, the spindle cell subtype presented with significantly smaller tumors (median 3.1 cm, IQR: 2.0–4.6 cm; *p* = 0.041). *Post-hoc* pairwise comparisons with Bonferroni correction showed a significant difference in tumor diameter between the spindle cell and mixed-type groups (adjusted *p* = 0.036), whereas no significant differences were observed between the spindle cell and squamous cell groups (adjusted *p* = 0.214) or between the squamous cell and mixed-type groups (adjusted *p* = 0.452). Detailed histopathological review of the 23 mixed-type cases showed chondroid-only metaplasia in 12 cases (52.2%), osseous-only metaplasia in 6 cases (26.1%), and combined chondroid and osseous metaplasia in 5 cases (21.7%). A radiological-pathological review showed that echogenic foci were seen in 6/6 osseous-only cases, 2/12 chondroid-only cases, and 3/5 combined chondroid and osseous cases. This finding may help explain the sonographic echogenic foci observed in these tumors.

### Sonographic features

The sonographic characteristics of the 50 lesions are detailed in Table 2. While the majority of tumors (74.0%, 37/50) exhibited an irregular shape, subgroup analysis revealed a distinct morphological pattern in the spindle cell subtype. Among these, 46.7% (7/15) of spindle cell carcinomas presented with a regular (oval or round) shape, frequently exhibiting a parallel orientation, and 60.0% (9/15) displayed circumscribed margins. In contrast, the squamous cell and mixed types typically manifested as irregular masses (83.3 and 82.6%, respectively; *p* = 0.093). Additionally, these two subtypes frequently displayed non-circumscribed margins (83.3 and 69.6%, respectively; *p* = 0.050). The pseudo-benign phenotype was identified in 12/50 tumors (24.0%). It was most frequent in the spindle cell subtype (8/15, 53.3%), compared with the squamous cell (1/12, 8.3%) and mixed-type groups (3/23, 13.0%) (overall Fisher’s exact test, *p* = 0.010, Cramer’s *V* = 0.452). Pairwise Fisher’s exact tests with Bonferroni correction showed that this phenotype was more frequent in spindle cell tumors than in mixed-type tumors (adjusted *p* = 0.035). The difference between spindle cell and squamous cell tumors did not remain significant after correction (adjusted *p* = 0.058), and no difference was found between squamous cell and mixed-type tumors (adjusted *p* = 1.000). Original ultrasound assessments showed low-suspicion initial assessment in 6 of 15 spindle cell lesions (40.0%), including BI-RADS 3 in 2 cases and BI-RADS 4A in 4 cases.

**Table 2:** Comparison of ultrasound imaging features among MBC subtypes.

Ultrasonic feature	Overall (*n* = 50)	Squamous cell (*n* = 12)	Spindle cell (*n* = 15)	Mixed type (*n* = 23)	*p*-value
Shape					
Irregular shape, *n* (%)	37 (74.0%)	10 (83.3%)	8 (53.3%)	19 (82.6%)	0.093^b^
Non-parallel orientation, *n* (%)	15 (30.0%)	4 (33.3%)	5 (33.3%)	6 (26.1%)	0.852^b^
Margin					
Circumscribed margin, *n* (%)	18 (36.0%)	2 (16.7%)	9 (60.0%)	7 (30.4%)	0.050^b^
Non-circumscribed margin^1^, *n* (%)	32 (64.0%)	10 (83.3%)	6 (40.0%)	16 (69.6%)	0.050^b^
Pseudo-benign phenotype, *n* (%)	12 (24.0%)	1 (8.3%)	8 (53.3%)	3 (13.0%)	0.010^c^*
Internal echo pattern					
Heterogeneous echogenicity, *n* (%)	31 (62.0%)	10(83.3%)	4 (26.7%)	17 (73.9%)	0.003^b^*
Complex cystic and solid, *n* (%)	26 (52.0%)	9 (75.0%)	4 (26.7%)	13 (56.5%)	0.037^b^*
Echogenic foci, *n* (%)	16 (32.0%)	2 (16.7%)	3 (20.0%)	11 (47.8%)	0.097^c^
Vascularity					
Hypervascularity (Grade II-III), *n* (%)	29 (58.0%)	8 (66.7%)	5 (33.3%)	16 (69.6%)	0.068^b^

^1^Non-circumscribed margin includes indistinct, angular, microlobulated, or spiculated margins. ^b^Chi-square test; ^c^Fisher’s exact test. *p < 0.05. For significant variables, pairwise *post-hoc* comparisons were performed with Bonferroni correction. Exact adjusted *p*-values are reported in the Results section.

Regarding internal echogenicity, the spindle cell subtype frequently demonstrated a homogeneous echo texture (73.3%, 11/15). Conversely, the squamous cell and mixed types frequently displayed heterogeneous echogenicity (83.3 and 73.9%, respectively; *p* = 0.003) or complex cystic and solid components (75.0 and 56.5%, respectively; *p* = 0.037). *Post-hoc* pairwise comparisons showed that heterogeneous echogenicity was significantly more common in squamous cell and mixed-type tumors than in spindle cell tumors (adjusted *p* = 0.021 and *p* = 0.023, respectively; squamous cell vs. mixed type, adjusted *p* = 1.000; overall Cramer’s *V* = 0.482). For complex cystic-solid components, although the frequency was higher in squamous cell and mixed-type tumors than in spindle cell tumors, the differences did not remain statistically significant after strict Bonferroni correction (adjusted *p* = 0.076 and *p* = 0.294, respectively; squamous cell vs. mixed type, adjusted *p* = 1.000; overall Cramer’s *V* = 0.359). Echogenic foci were present in 32.0% of all cases, with the highest prevalence observed in the mixed type (47.8%, 11/23), although this trend did not reach statistical significance (*p* = 0.097). Hypervascularity (Adler grade II-III) was common (58.0%) across all subtypes, with no significant intergroup differences (*p* = 0.068).

### Tumor size and lymph node correlation

The overall rate of axillary lymph node metastasis in the cohort was 22.0% (11/50). Despite the significant heterogeneity in tumor size noted previously, no statistically significant difference in nodal positivity was observed among the pathological subtypes (*p* = 0.514). For instance, although the mixed type presented with the largest tumor burden (mean 5.6 cm), its nodal metastasis rate (21.7%, 5/23) was not significantly higher than that of the much smaller spindle cell carcinoma (13.3%, 2/15).

Nodal involvement differed numerically across tumor-size groups but did not show a monotonic increase (Figure 1B). The nodal metastasis rates were 0.0% in tumors ≤2 cm (0/6), 26.1% in tumors measuring 2–5 cm (6/23), and 23.8% in tumors >5 cm (5/21). This difference was not statistically significant by the Fisher–Freeman–Halton exact test (*p* = 0.563), and no significant linear trend was detected across ordered size categories (*p* = 0.387). Because only 11 nodal metastasis events were observed, multivariable modeling was not performed. Therefore, the observed size-node pattern should be interpreted as a descriptive and hypothesis-generating finding, not as evidence of a size-independent metastatic mechanism.

## Discussion

In this cohort of 50 patients, primary metaplastic breast cancer (MBC) subtypes demonstrated distinct sonographic and clinicopathological patterns (17, 18). The spindle cell subtype frequently presented with a pseudo-benign sonographic appearance (17, 19), whereas the squamous cell and mixed type exhibited more complex sonographic features, characterized by frequent cystic-solid components and heterogeneous echogenicity, with echogenic foci particularly observed in mixed-type tumors (9, 18, 20). In addition, a descriptive pattern suggestive of dissociation between tumor size and nodal status was observed, although this finding requires validation in larger cohorts with outcome data (12, 21).

**Figure 2:**
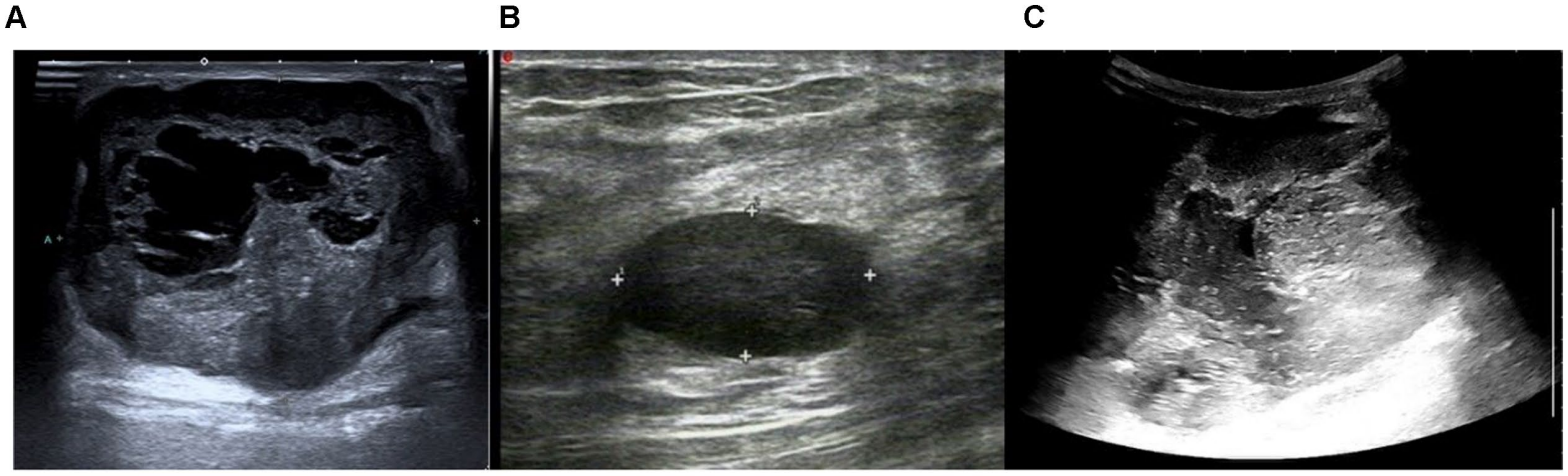
**(A)** Squamous cell carcinoma: Ultrasound image of an 80-year-old woman reveals a large, predominantly solid mass measuring 5.5 × 4.2 cm. The tumor displays an irregular shape with indistinct, angular margins. The focal anechoic area is suggestive of cystic degeneration or necrosis. **(B)** Spindle Cell Carcinoma: Ultrasound image of a 56-year-old woman presenting with a 2.1 × 1.2 cm mass. The lesion exhibits benign-appearing features, including a regular oval shape, circumscribed margins, and a parallel orientation. The internal echo texture is homogeneous and hypoechoic, closely mimicking a fibroadenoma. This pseudo-benign appearance can lead to initial diagnostic underestimation, as shown by some spindle cell lesions being classified as BI-RADS 3 or 4A. **(C)** Mixed type: Ultrasound image of a 42-year-old woman with a giant 11.2 cm mass. The tumor presents as a lobulated mass with microlobulated margins, reflecting its expansive growth pattern. It appears as a predominantly solid, heterogeneous mass with a complex internal architecture and scattered punctate or patchy echogenic foci.

Sonographically, 60.0% of the evaluated spindle cell tumors displayed well-circumscribed margins, and 46.7% showed a regular shape, contrasting with typical signs of breast malignancy. This benign-like appearance is likely explained by the expansile, “pushing” growth pattern of spindle cell carcinomas rather than an infiltrative one. These tumors also lack a pronounced desmoplastic stromal reaction, which contributes to the absence of classic spiculated margins commonly seen in invasive ductal carcinomas (9). Due to this deceptive appearance, 6 of 15 spindle cell lesions (40.0%) were initially assigned to low-suspicion BI-RADS categories, either BI-RADS 3 or 4A (Figure 2B). BI-RADS 4A usually prompts biopsy, but it still reflects a low level of suspicion. Therefore, classification as BI-RADS 3 or 4A in pathologically confirmed spindle cell MBC is better understood as diagnostic underestimation rather than benign misdiagnosis. This finding supports prompt biopsy for rapidly growing solid lesions with benign-looking features (22). To overcome this morphological overlap, recent advances in radiomics have demonstrated that extracting sub-visual quantitative features can effectively distinguish metaplastic histology at initial diagnosis, thereby improving preoperative diagnostic accuracy and preventing ineffective interventions (23).

Unlike the spindle cell variants, the squamous and mixed types more frequently displayed overtly malignant sonographic features. In our cohort, 75.0% of squamous cell carcinomas appeared as complex cystic and solid masses. This complex morphology may correspond to cystic degeneration, hemorrhage, or necrosis on pathology (9). A possible explanation is that rapid tumor growth may outstrip the local blood supply, but this mechanism was not directly tested in the present cohort. Intense neoangiogenesis, reflected by frequent Adler grade II–III hypervascularity, is observed in these tumors. However, rapid tumor proliferation may outstrip the local blood supply, potentially contributing to central necrosis and liquefaction. Clinically, such extensive necrosis can trigger severe localized inflammation, mimicking acute breast abscesses or inflammatory breast cancer. Meanwhile, the mixed type frequently produces bone or cartilage matrix. Echogenic foci may reflect calcifications or osseous/chondroid matrix components and may provide a useful sonographic clue for mixed-type tumors (20).

A notable pattern observed in this study was the dissociation between tumor size and nodal involvement, where the overall axillary metastasis rate remained relatively stable (22.0%) despite a large mean tumor size of 4.7 cm. The metastasis rate remained relatively stable in tumors larger than 2 cm (26.1% for 2–5 cm; 23.8% for >5 cm). This observation aligns with population-based analyses indicating that MBCs often present with lower regional nodal involvement compared to standard invasive ductal carcinomas (12, 21, 24). This pattern has been hypothesized to be related to epithelial–mesenchymal transition biology, which may favor hematogenous spread (14). Recent molecular studies have reported frequent alterations in the PI3K/AKT/mTOR pathway, EGFR overexpression, tumor-infiltrating lymphocytes, and PD-L1 expression in MBC (5, 25). These findings provide biological context for the aggressive phenotype of MBC. However, the present cohort did not include molecular profiling, so direct correlations between these molecular alterations and ultrasound features cannot be established. Early ultrasound suspicion may help prompt tissue diagnosis, after which pathological and molecular testing can guide systemic therapy.

Clinically, these subtype-specific features inform several practical management strategies. To mitigate diagnostic delays for spindle cell carcinomas, our findings support the need for prompt biopsy in rapidly enlarging solid lesions, even when benign morphological features are present (26). Furthermore, when biopsying squamous and mixed types, sampling should specifically target the peripheral viable tissue to avoid non-diagnostic yields from necrotic debris. Finally, early ultrasound recognition of these aggressive subtypes may help inform treatment timing. These sonographic findings should not independently determine treatment strategy, but they may support earlier tissue diagnosis and multidisciplinary discussion. In operable tumors with sonographic features suggestive of aggressive squamous or mixed-type MBC, upfront surgery may be considered rather than prolonged standard neoadjuvant chemotherapy, given the reported low pathological complete response rates of MBC to standard neoadjuvant regimens (4, 5). Accordingly, imaging findings should be interpreted as supportive evidence within comprehensive multidisciplinary management.

This study has several limitations. First, this was a single-center retrospective study, and residual selection bias could not be fully excluded despite the inclusion of all eligible consecutive patients. The long study period may also have introduced temporal heterogeneity in ultrasound equipment, BI-RADS use, and clinical practice. Although all available images were re-evaluated using the BI-RADS 5th edition lexicon, residual variability may remain. Second, no formal *a priori* sample size or power calculation was performed because of the rarity of MBC and the consecutive-case design. The small sample size, especially within each pathological subtype, limited statistical power and precluded reliable multivariable analysis. Therefore, our findings should be interpreted as exploratory. Third, several ultrasound features were compared. Although Bonferroni correction and effect sizes were used when appropriate, type I error cannot be fully excluded. Finally, long-term survival, recurrence, and distant metastasis data were not available. Therefore, this study cannot directly link ultrasound subtypes with prognosis. Future multicenter studies with standardized follow-up are needed.

In conclusion, MBC demonstrates substantial heterogeneity in both ultrasound features and baseline clinicopathological characteristics. The pseudo-benign appearance of the spindle cell subtype remains a major diagnostic pitfall, particularly in rapidly enlarging solid lesions. The observed size-node pattern should be interpreted as descriptive and hypothesis-generating, and it requires validation in larger cohorts with longitudinal outcome data. These findings may improve diagnostic awareness and support more timely multidisciplinary management.

## Data Availability

The datasets presented in this article are not readily available. The ethical approval granted for this retrospective study does not include authorization for data distribution to third parties.
